# Extracapillary proliferation in IgA nephropathy; recent findings and new ideas

**DOI:** 10.12860/jnp.2015.01

**Published:** 2015-01-01

**Authors:** Hamid Nasri, Muhammed Mubarak

**Affiliations:** ^1^Department of Nephrology, Division of Nephropathology, Isfahan University of Medical Sciences, Isfahan, Iran; ^2^Department of Histopathology, Sindh Institute of Urology and Transplantation (SIUT), Karachi, Pakistan

**Keywords:** IgA nephropathy, Oxford classification, Extracapillary proliferation, Crescents, End-stage renal disease

## Abstract

*Context:* IgA nephropathy (IgAN) is an autoimmune disorder and is the most common form of primary glomerulonephritis (GN) worldwide.

*Evidence Acquisitions:* Directory of Open Access Journals (DOAJ), Google Scholar, PubMed (NLM), LISTA (EBSCO) and Web of Science have been searched.

*Results:* It is a slowly progressing disorder that leads to end-stage renal disease (ESRD) in up to 50% of the patients within 25 years of the onset of the disease. IgAN is defined by predominant IgA deposition in the mesangial area on immunofluorescence (IF) microscopy. Its histology varies from mild focal segmental proliferation of mesangial cells to severe diffuse global proliferation with extracapillary proliferation (crescent formation). The Oxford classification, designed in 2009, is a new classification for the evaluation of morphologic lesions of IgAN. This classification, containing four pathology variables, was found to have prognostic implications. The variables included are mesangial hypercellularity (M), endocapillary proliferation (E), segmental glomerulosclerosis (S) and the proportion of interstitial fibrosis and tubular atrophy (T). However, crescents were not included in the Oxford classification.

*Conclusions:* In this mini-review, we describe the recent publications about the significance of extracapillary proliferation in IgAN and we conclude that, there is much controversy about the role of extracapillary proliferation as a significant prognostic factor in IgAN. Hence, it is important to re-consider crescents in IgAN patients. Therefore, we suggest further investigations on this aspect of IgAN disease.

**Implication for health policy/practice/research/medical education:**

There is still controversy about the role of extracapillary proliferation as a significant prognostic factor in IgAN. It is important to conduct more studies on the value of crescents in IgAN patients on the pattern adopted for the development of the original classification before these lesions are included in the Oxford classification of IgAN.

## 1. Context

### IgA nephropathy: Diagnosis and prognosis

Immunoglobulin A nephropathy (IgAN) is an autoimmune disorder and is the most common form of primary glomerulonephritis (GN) worldwide (1-5). IgAN is a slowly progressing disorder that leads to end-stage renal disease (ESRD) in up to 40% of the patients within 25 years of the diagnosis (6,7). IgAN is defined by predominant IgA deposition in the mesangial area on immunofluorescence (IF) microscopy. Its histology varies from mild focal segmental proliferation of mesangial cells to severe diffuse global proliferation and extracapillary proliferation (crescent formation) in many cases (1-5).

## 2. Evidence Acquisition

Directory of Open Access Journals (DOAJ) Google Scholar, PubMed (NLM), LISTA (EBSCO) and Web of Science were searched with key words relevant to IgA nephropathy, Oxford classification, Extracapillary proliferation, Crescents, End-stage renal disease.

## 3. Results

Various factors are associated with the accelerated progression of this glomerulopathy leading to early development of ESRD (8). Clinical factors, such as, persistent hematuria or proteinuria, elevated serum creatinine and poorly controlled hypertension, are the known risk factors for deterioration of IgAN. The progression of the disease may be delayed if the associated risk factors are controlled (9). The prognostic value of pathological features on renal biopsies has been controversial till recent past. The Oxford classification, designed in 2009, is a new classification for the evaluation of morphologic lesions as independent prognosticators of IgAN (10,11). This classification has reasonable reproducibility and can be used to assess the long-term prognosis and response to treatment. This classification containing four pathology variables has been widely validated to have prognostic implications. The pathology variables are; a) mesangial hypercellularity (M), b) endocapillary proliferation (E), c) segmental glomerulosclerosis (S) and d) the proportion of interstitial fibrosis and tubular atrophy (IF/TA) (T). The value of extracapillary proliferation (crescents) was not addressed in the original study, mainly due to the scarcity of the lesion in the study cohort and the exclusion of rapidly progressive forms of the disease (10,11). However, studies by other investigators have addressed the significance of this lesion in other cohorts with variable results (12-22). This mini-review is intended to record the recent studies exploring the significance of extracapillary proliferation (crescents) in IgAN.

## 4. Extracapillary proliferation (crescents) in IgA nephropathy

**Figure 1:**
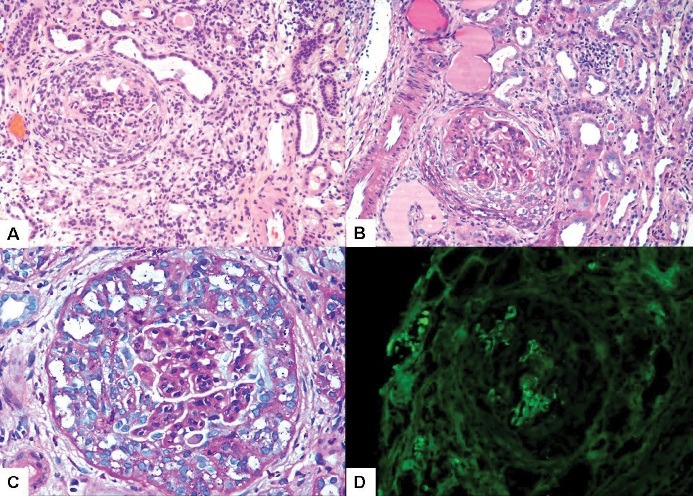
Crescentic IgA nephropathy. A. Medium-power view showing a cellular crescent in one glomerulus. Surrounding parenchyma shows moderate chronic interstitial inflammation and marked acute tubular injury. (Hematoxylin and Eosin, ×200). B. Another glomerulus showing crescent with early organization. There is interstitial inflammation in the surrounding parenchyma. (Periodic acid-Schiff, ×200). C. High-power view showing a full-blown cellular crescent. (Periodic acid-Schiff, ×400). D. Immunofluorescence staining showing 2+ positivity of immunoglobulin (Ig) A in the collapsed glomerular tufts. Surrounding crescent is negative. (Immunofluorescence for IgA, ×200)

The value of extracapillary proliferation (crescents) was not assessed in the original study leading to the formulation of the Oxford classification of IgAN, primarily because of scarcity of the lesion in the study cohort (10,11). This was due to the selective inclusion criteria used for selecting the study subjects. However, the authors of the original study stressed the need for further corroborative and validation studies in other cohorts and including more diverse morphological lesions (10). Crescentic IgA glomerulonephritis, defined as >50% crescentic glomeruli on renal biopsy (Figure 1), is one of the causes of rapidly progressive glomerulonephritis (RPGN) (1). It is well known that, if extracapillary proliferation of whatever etiology is not treated, it will lead to rapid progression to ESRD (8). However, few investigations have primarily addressed this lesion in IgAN patients. A few investigators have attempted to correlate the extracapillary proliferation in IgAN with clinical outcomes in different study cohorts. These have produced conflicting results. In fact, as is well known, IgAN is a highly heterogeneous disease with variable clinical patterns, morphologic variables, long-term renal progression, and geographic prevalence (1-5). Many studies have proven that extracapillary proliferation may have prognostic significance (12-14). Recently, we investigated the clinical significance of crescents in a group of IgAN patients (15). Our study was a cross-sectional and observational investigation. We collected a total of 114 (70.2%; male) biopsies. We diagnosed IgAN by light and immunofluorescence (IF) microscopy. The diagnosis of IgAN was based on prominent (≥2+) IgA deposits in mesangial area with negative C1q deposits. The mean age of the patients was 37.7±13.6 years. The mean serum creatinine was 1.6±1.5 mg/dl. Twenty-five (21.9%) patients had crescents on renal biopsies. We found a significantly positive correlation between the proportion of crescents and serum creatinine (p<0.001). Additionally, we observed a positive correlation between nephrotic-range proteinuria and the proportion of extracapillary proliferation (p<0.05). Furthermore, a significantly positive correlation between the proportion of globally sclerotic glomeruli and extracapillary proliferation (p=0.028) was detected. These findings demonstrate that crescents have a significant association with proteinuria and proportion of globally sclerotic glomeruli (15). Katafuchi *et al*. conducted a study to find out the significance of pathologic features for development of ESRD by multivariate analysis in 702 patients with IgAN (16). They examined the correlation of crescents with kidney survival by univariate analysis in 416 patients who met the Oxford criteria and 286 who did not, separately. In a multivariate model, S and T variables of the Oxford classification were significantly correlated with the development of ESRD. They also found that extracapillary proliferation was significant for the development of ESRD. In univariate analysis, kidney survival was significantly lower in patients with crescents than in those without. They clearly demonstrated the prognostic significance of crescents in their patients and suggested that crescents be included in the next revision of the Oxford classification of IgAN to widen the scope of the classification (16). In the study of Walsh *et al*., on 146 patients with IgAN with median follow-up of 5.8 years, with primary outcomes of doubling serum creatinine, ESRD or death, they found that the clinical and histopathological predictors of outcome were initial serum creatinine, proteinuria, systolic blood pressure, glomerulosclerosis, IFTA and crescentic disease on univariate analysis (17). On multivariate analysis adjusted for clinical characteristics, histopathological variables of IF/TA, glomerulosclerosis and crescentic disease remained independent predictors of the primary outcome (17). Likewise, Choi *et al*., (18) conducted an investigation to detect the prognostic relevance of clinical and morphologic features on renal outcome in patients with IgAN treated with the combination therapy of steroids and angiotensin receptor blockers (ARBs). Regression analysis revealed that the final urine protein/creatinine excretion ratio and age were critical elements of slope of the estimated glomerular filtration rate (eGFR) by both univariate and multivariate analysis. Surprisingly in their study, eGFR, systolic blood pressure, proteinuria, and body mass index (BMI) at the initial presentation were not predictive of slope. They also found that no histological feature, including crescents, predicted slope. They concluded that, achieving a low urinary protein excretion is the main determinant for the good outcome in patients treated with combination therapy (18).

To identify the risk factors and develop a prediction model, Lv *et al*. assessed data from patients ≥14 years old with crescentic IgAN who were followed for ≥12 months (19). They examined 52 patients from one renal center (discovery cohort), and the validation cohort comprised 61 patients from multiple centers. At biopsy, the mean serum creatinine level was 4.3±3.4 mg/dl, and the mean percentage of crescents was 66.4±15.8 %. The renal survival rates at years 1, 3, and 5 after biopsy were 57.4±4.7%, 45.8±5.1%, and 30.4±6.6%, respectively. Multivariate Cox regression disclosed initial serum creatinine level as the only independent risk factor for ESRD. Notably, the percentage of crescents did not associate independently with ESRD. Logistic regression showed that the risk of ESRD at 1 year after biopsy increased rapidly at serum creatinine >2.7 mg/dl and reached 90% at serum creatinine >6.8 mg/dl. In both cohorts, patients with serum creatinine >6.8 mg/dl were less probable to recover from dialysis. They concluded that crescentic IgAN has a poor prognosis and initial serum creatinine concentration may predict kidney failure in patients with this disease. Additionally, a recent systematic review and meta-analysis was conducted by Lv *et al*, on patients with biopsy-proven IgAN by assessing the Oxford classification (20). In their review, they defined kidney failure as doubling of serum creatinine level, 50% decline in eGFR, or ESRD. They considered 16 retrospective cohort studies with 3,893 patients and 570 kidney failure events. In a multivariate model, hazard ratios for renal failure were 0.6, 1.8, and 3.2 for scores of M0 (mesangial hypercellularity score ≤0.5), S1 (presence of segmental glomerulosclerosis), and T1/2 (>25% IFTA), respectively, without evidence of heterogeneity (20). They showed that, endocapillary proliferation (E) lesions were not associated with kidney failure, with evidence of heterogeneity. They also found that extracapillary proliferation was associated with kidney failure, with no evidence of heterogeneity. They finally concluded that, mesangial hypercellularity (M), segmental glomerulosclerosis (S), the proportion of IFTA (T), and extracapillary proliferation, but not endocapillary proliferation (E) lesions, are correlated intensely with progression to kidney failure and thus should be included in the Oxford-MEST system (20). Interestingly, Lee *et al*, on a total of 430 patients with biopsy-proven IgAN between January 2000 and December 2009, assessed the morphologic variables of the Oxford classification including the presence of extracapillary proliferation (21,22). They found that 18.8% had crescents on renal biopsies. During a mean follow-up of 61 months, the primary outcome occurred in 23.5% of patients with extracapillary proliferation compared with 11.5% patients without extracapillary proliferation. The 10-year renal survival rate was significantly lower in patients with crescents than those without crescents. However, in a multivariable Cox analysis which included clinical factors and the Oxford classification, crescents were not significantly associated with an increased risk of developing the primary outcome. They concluded that, adding extracapillary proliferation to the Oxford classification did not improve the discriminative ability for the prediction of renal outcomes. They interpreted that crescents were not independent prognostic factor, suggesting that crescents have limited value in predicting kidney outcomes of IgAN (21,22).

It is evident from above that extracapillary proliferation is of prognostic importance in IgAN. However, there is no complete agreement on this and more studies are needed to clarify its role before this lesion can be formally included in the Oxford classification. There is also a need for search for new molecular genetic and omics markers to further improve the histopathological classification for prognostication and individualized treatment of patients with IgAN (23).

## 5. Conclusions

There is still controversy about the role of extracapillary proliferation as a significant prognostic factor in IgAN. It is important to conduct more studies on the value of crescents in IgAN patients on the pattern adopted for the development of the original classification before these lesions are included in the Oxford classification of IgAN.

## Authors’ contributions

All authors wrote the manuscript equally.

## Conflict of interests

The authors declared no competing interests.

## Funding/Support

None.
